# Metabolomics Reveals the Allelopathic Potential of the Invasive Plant *Eupatorium adenophorum*

**DOI:** 10.3390/plants10071473

**Published:** 2021-07-19

**Authors:** Xunzhi Zhu, Yangmin Yi, Ling Huang, Chi Zhang, Hua Shao

**Affiliations:** 1 Institute of Botany, Jiangsu Province and Chinese Academy of Sciences, Nanjing 210014, China; zhuxunzhi@cnbg.net; 2 State Key Laboratory of Hydrology-Water Resources and Hydraulic Engineering, Hohai University, Nanjing 210098, China; 3 College of Biotechnology, Jiangsu University of Science and Technology, Zhenjiang 212018, China; 179310005@stu.just.edu.cn; 4 State Key Laboratory of Desert and Oasis Ecology, Xinjiang Institute of Ecology and Geography, Chinese Academy of Sciences, Urumqi 830011, China; huangling201@mails.ucas.ac.cn; 5 Shandong Provincial Key Laboratory of Water and Soil Conservation and Environmental Protection, College of Resources and Environment, Linyi University, Linyi 276000, China

**Keywords:** allelopathy, exotic plant invasion, metabolomics, phosphate uptake, physiological mechanism

## Abstract

Phytotoxic chemicals produced by alien invasive plants exert inhibitory effects on native species to facilitate their invasiveness. The allelopathic process of invaders has been hypothesized as the “Novel Weapon Hypothesis”. However, this hypothesis has been controversial for decades due to lack of molecular evidence, and the underlying mechanism of allelopathy still remains ambiguous. Herein, we explore the allelopathic mechanisms of *Eupatorium adenophorum*, a world-widely spread noxious weed, by the methods of laboratory bioassay and metabolomics analyses in the recipient plant, *Arabidopsis thaliana*. The bioassay revealed significant phytotoxicity of *E. adenophorum* extracts. A total of 234 metabolites in *A. thaliana* were detected by Gas Chromatographic−Mass Spectrometric analysis. There were 48, 99 and 94 impacted metabolites in *A. thaliana* treated by 50, 25 and 12.5% aqueous extracts compared to control. When mapping all the impacted metabolites to the biological pathways in the KEGG (Kyoto Encyclopedia of Genes and Genomes) database, we found mineral absorption, ABC transporters, amino acid biosynthesis, metabolic pathways and biosynthesis of plant secondary metabolites were mainly impacted. Synthesized with partial least-squares discriminate analysis (PLS-DA) results of metabolic profiles in *A. thaliana*, we found that citrate cycle was suppressed, metabolism of amino acids was disordered and phosphate absorption was inhibited. Subsequent investigation demonstrated that the phosphorus content in *A. thaliana* tissues exposed in allelopathic extracts was much lower, indicating inhibition of phosphate uptake. Our study revealed by metabolomics approaches that *E. adenophorum* is an allelopathic species.

## 1. Introduction

*Eupatorium adenophorum* Spreng. (Syn. *Ageratina adenophora* (Spreng.) King & H. Rob.) is a worldwide aggressive invasive weed, which is a perennial herb or subshrub native to Central America. Currently, *E. adenophorum* is widely distributed in many countries and regions in Asia, Europe, Oceania and Africa [1,2], and has significant impact on local ecosystems, agriculture and husbandry, and causes a serious threat to human and animal health. Its invasion can form monodominant communities, affect the growth of native plants, and reduce the diversity of native species [3,4]. The “Novel Weapons Hypothesis”, which was created by Callaway and Ridenour [5], is one of the most important theories to explain the mechanisms of this successful invasion. The main idea is that *E. adenophorum* can negatively affect native plants by releasing novel allelochemicals and crowd them out. About two decades ago, researchers found that the invasiveness of *E. adenophorum* had a close relationship with its secondary metabolites [6], and the chloroform extract of aerial parts could inhibit seed germination and seedling growth of onion (*Allium cepa* L.), radish (*Raphanus sativus* L.) and cucumber (*Cucumis sativus* L.) [7]. Yang et al. [8] isolated and purified two substances from the aqueous extract of *E. adenophorum* stems and leaves that were demonstrated to pose significant allelopathic effects to other plant species. The two allelochemicals were thereafter named as 9β-Hydroxyageraphorone (HHO) and 9-Oxo-10,11-dehydroageraphorone (DTD or ODA); the latter was also known as Euptox A. These two allelopathic substances can cause changes in the activities of malondialdehyde and peroxidase in rice (*Oryza sativa* L.) root cells, as well as imbalance in physiological and biochemical indicators such as abscisic acid (ABA) and indoleacetic acid (IAA) [8,9]. Because they are found only in *E. adenophorum*, they were thought to be novel compound weapons that could impact native plants. Leaching by rain or fog drip into the soil was thought to be the main method of release. *E. adenophorum* was shown to produce a suite of allelochemicals in the leaves and roots [10,11] and the allelopathic potential of leaf and root leachate on native species has been documented [12,13]. Subsequently, Yang et al. [14] isolated and identified root exudates of *E. adenophorum* in which Euptox A was identified. Yang et al. found that allelopathic compounds of *E. adenophorum* could degrade and maintain very low concentrations in soil (much lower than people used in laboratory bioassays), but the trace allelochemicals could also exhibit phytotoxic effects when they came together [15]. In addition to the allelochemicals mentioned above, four kinds of quinic acid derivatives were extracted and identified from aerial parts of *E. adenophorum* and their bioactivity were demonstrated [16].

Previously, laboratory bioassays have been mainly used to study *E. andenophorum* toxicity; however, such studies lack the molecular mechanism of allelopathy. This situation makes the chemical weapon hypothesis on the allelopathic mechanism of plant invasion controversial. How allelochemicals of invasive plants alter the metabolic pathways of native plants, affecting their physiological and phenotypic characteristics, and ultimately their competitive advantage and fitness, is still poorly understood [17,18]. In this study, we aimed to reveal the allelopathic potential of *E. adenophorum* by both laboratory bioassay and metabolomics analyses. As allelopathic chemicals are usually naturally released from plant aerial parts by dew, rain or fog drip, aqueous extracts of *E. adenophorum* were used to assess allelopathic potential. Based on metabolomics results, a further study was conducted aiming to verify the metabolomics analyses and reveal the underlying physiological mechanism of allelopathy.

## 2. Results

### 2.1. In Vitro Bioassay

**Figure 1:**
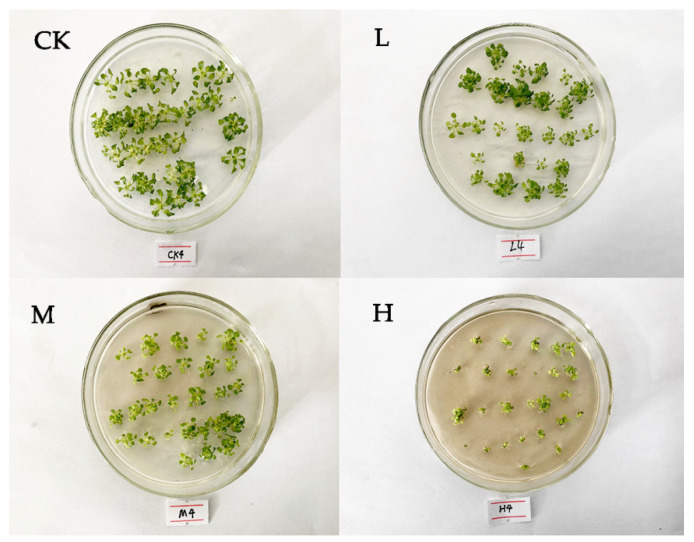
Growth status of 27-day *Arabidopsis thaliana* treated with *Eupatorium adenophorum* aqueous extracts in Petri dishes. Abbreviations: CK—control; L—treated by aqueous extract in low concentration; M—treated by aqueous extract in medium concentration; H—treated by aqueous extract in high concentration.

**Figure 2:**
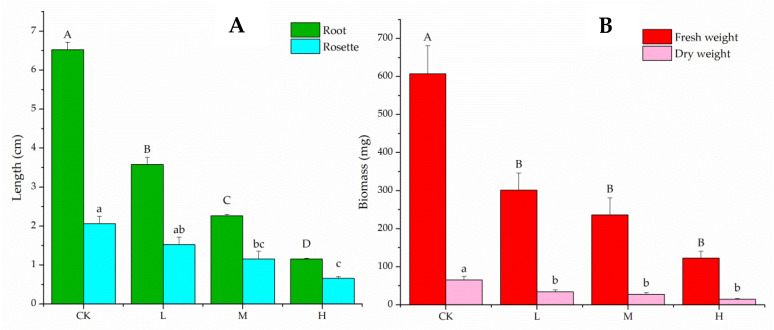
Means and SE values are shown for effects of *Eupatorium adenophorum* aqueous extracts on root and rosette leaf length (**A**), and biomass (**B**) of *Arabidopsis thaliana*. Abbreviations: CK–control; L–medium with low concentration extract; M–medium with medium concentration extract; H–medium with high concentration extract. Bars with different letters indicate significant differences at *p* < 0.05 (Tukey′s HSD test).

*Eupatorium adenophorum* extracts induced overt toxicity on *Arabidopsis thaliana* seedlings. Compared to the control group (abbreviated as CK), *A. thaliana* treated with three concentrations of aqueous extract (low concentration aqueous extract, medium concentration aqueous extract and high concentration aqueous extract, abbreviated as L, M and H) all showed significant morphological differences (Figure 1). *A. thaliana* grew smaller in the high concentration extract (H group) than in the medium and low concentration extracts (M and L groups). Besides, the high concentration aqueous extract (H treatment) induced leaf yellowing, indicating leaf chlorotic damage and senescence (Figure 1). With the increase of the concentration of *E. adenophorum* extract, the root length and the rosette leaf length of *A. thaliana* decreased significantly (Figure 2A). When compared to the control (CK), *A. thaliana* root length was reduced by 45% in the low concentration extract treatment, by 65% (L) in the medium concentration extract (M) and by 82% in the high concentration extract The biomass, both fresh weight and dry weight, of *A. thaliana* seedlings, showed a significant decrease when exposed in *E. adenophorum* extracts (Figure 2B). The fresh weight in the low concentration of extract was about 50% lower compared to the control (CK), while that in medium and high concentration of extract was lower by about 61% and 75%, respectively. After exposure to the L solution, the dry weight of *A. thaliana* was 52% of the control, and reduced to 42% and 22% after exposure to the M and H solutions, respectively.

### 2.2. Metabolite Analysis in Arabidopsis thaliana

**Figure 3:**
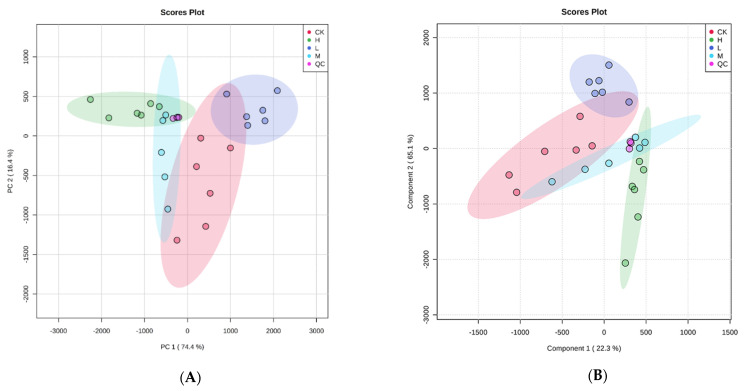
Principal component analysis (PCA) (**A**) and partial least-squares discriminate analysis (PLS-DA) score plots (**B**) of metabolic profiles in *Arabidopsis thaliana* treated with *Eupatorium adenophorum* aqueous extracts. Abbreviations: CK—control; L—treated by aqueous extract in low concentration; M—treated by aqueous extract in medium concentration; H—treated by aqueous extract in high concentration; QC—quality control, i.e., the sample prepared by mixing aliquots of all samples.

PCA (Figure 3A) showed that L, M, and H were significantly separated from the control group along with the first principal component (PC1), accounting for 74.4% of the total variance. In the PCA plot, various treatments (L, M, and H) were separated. The score chart of PLS-DA (Figure 3B) showed that all the treatment groups were significantly separated from the control group. These results indicate that different concentrations of *E. adenophorum* extracts changed the metabolic characteristics of the recipient plant, *A. thaliana*, differently.

**Figure 4:**
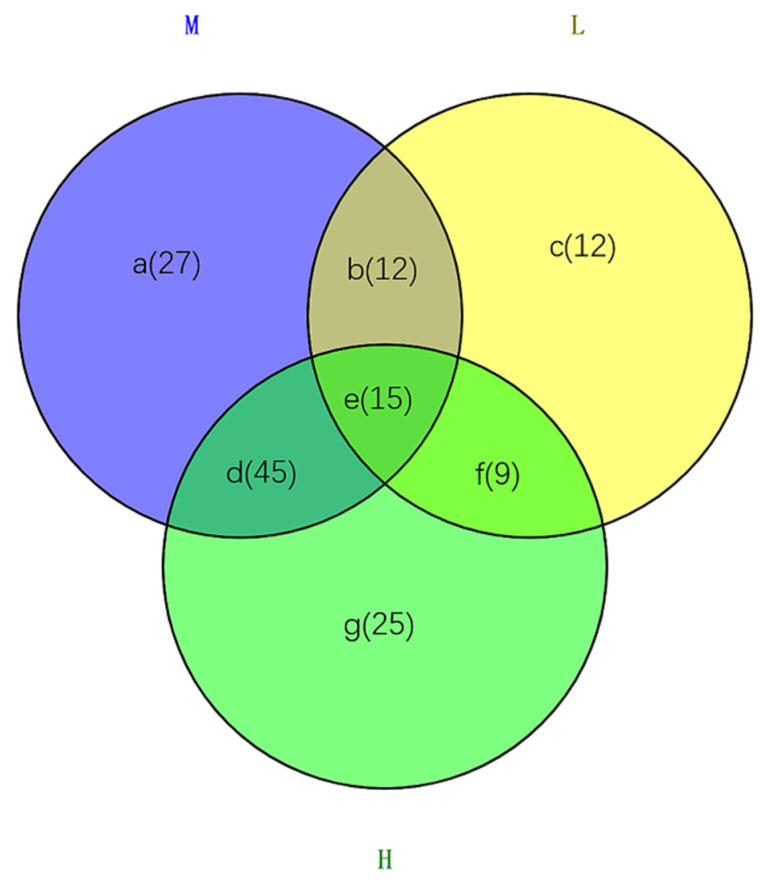
Venn plot for number of differential metabolites impacted by L, M and H extract solutions. The overlaps of two of the three circles indicate the number of metabolites impacted by the two. The overlap of the three circles indicates the number of metabolites all impacted by three solutions. The letters a to g represent different parts of the Venn plot. Numbers in parentheses represent the number of metabolites. Details of the names and changes of metabolites can be seen in Table S1 in Supplementary.

According to the VIP (variable importance in projection) score > 1 criteria, a total of 145 different compounds were found when comparing L to CK, M to CK and H to CK. Among them, 48 significantly changed metabolites were found between the groups of L and CK, 99 significantly changed metabolites between the groups of M and CK, and 94 between the groups of H and CK (Figure 4). There were 12 unique differently regulated metabolites between L and CK, suggesting that these 12 metabolites were not significantly impacted when treated with medium concentration and high-concentration aqueous extracts (M and H). Similarly, there were 27 and 25 different metabolites affected by M and H extract solutions (Figure 4). However, there were 15 metabolites all impacted by three different concentration extracts. All the 145 differential metabolites detected above the threshold are shown in Table S1. In these metabolites, a total number of 80 compounds were upregulated (i.e., increased), 44 compounds were downregulated (i.e., decreased), and others had no consistent regulation under different treatments. In this table it is shown that amino acids, sugars, and organic acids had the most variety (Table S1).

**Figure 5:**
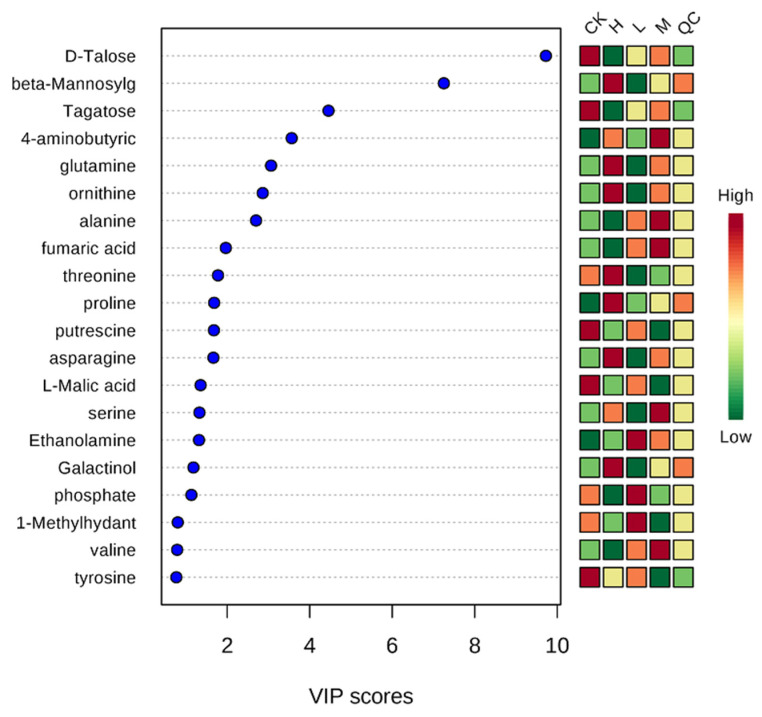
Variable importance in projection (VIP) scores from partial least-squares discriminate analysis (PLS-DA) of metabolic profiles in *Arabidopsis thaliana* treated with *Eupatorium adenophorum* aqueous extracts. Abbreviations: CK–control; L–treated by aqueous extract in low concentration; M–treated by aqueous extract in medium concentration; H–treated by aqueous extract in high concentration; QC–quality control, i.e., the sample prepared by mixing aliquots of all samples.

According to PLS-DA results, the first 20 metabolites that led to the separation between the control group and the three treatment groups are shown in Figure 5. Within the 20 metabolites, a total of 12 compounds were amino acids, such as alanine, valine, asparagine, threonine, serine, glutamine, and others. Others were carbohydrates ad organic acids. It is notable that malic acid is in this figure, which is the conjugate acid of a malate. As we know, malate is a key compound in the citrate cycle (TCA cycle), which is the most important pathway of plant energy metabolism. Phosphate, the only inorganic substance, is also in Figure 5, which implies that phosphate absorption was affected by allelopathy. Thus, an experiment on phosphate absorption of *A. thaliana* was conducted, and the results were presented below.

**Figure 6:**
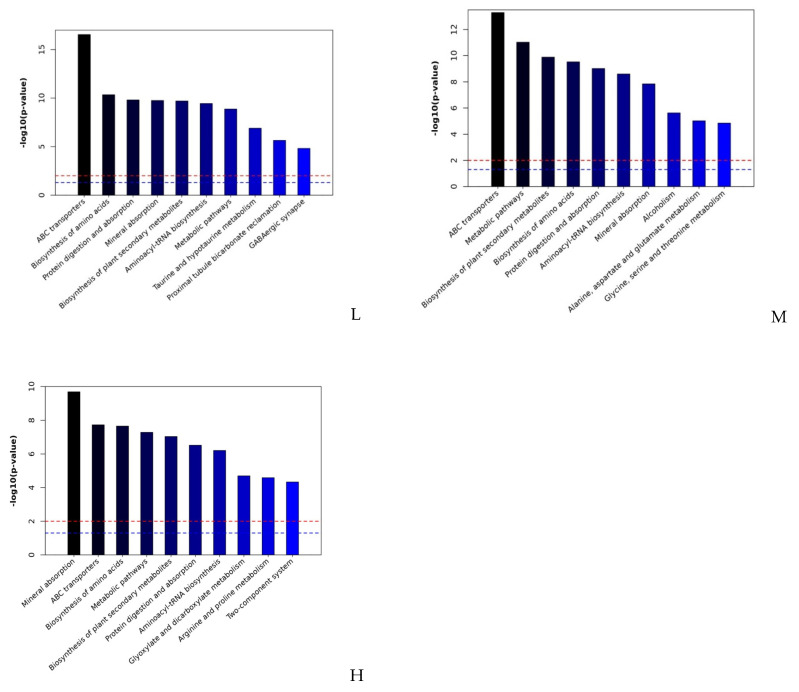
Histogram of the significances of biological pathways. Fisher’s exact test was performed to obtain the *p* value. The red dashed line represents *p* = 0.01. The blue dashed line represents *p* = 0.05. Data are from the impacted metabolites of *A. thaliana* treated by low concentration extract of *E. adenophorum* (L), medium concentration extract of *E. adenophorum* (M), and high concentration extract of *E. adenophorum* (H).

The results above indicated that amino acid dynamics, energy metabolism and phosphate absorption were mostly responsible for allelopathic effects of *E. adenophorum*. Here all the impacted metabolites affected by the stress of *E. adenophorum* extracts were mapped to the biological pathways involved in the Kyoto Encyclopedia of Genes and Genomes (KEGG) database and were respectively assigned to 152 pathways [19]. Compared with the KEGG pathway library of *Arabidopsis thaliana*, different metabolites were enriched in ten main pathways. The logarithms of *p* values obtained from Fisher’s exact test of the 10 metabolic pathways’ enrichment are showed in Figure 6. Mineral absorption, ABC transporters, amino acid biosynthesis, metabolic pathways and biosynthesis of plant secondary metabolites were the main impacted pathways (Figure 6).

**Figure 7:**
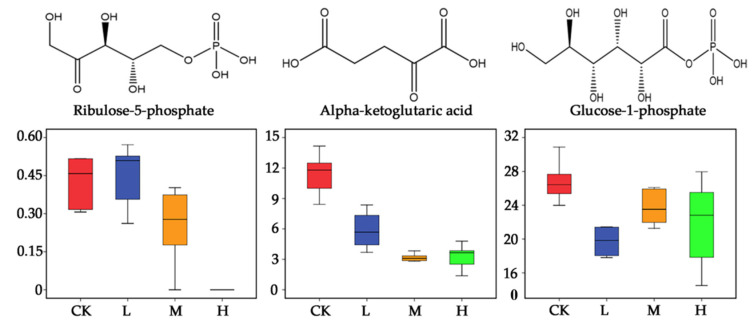
Box plots of relative abundance of energy–related metabolic chemicals of *Arabidopsis thaliana* treated with aqueous extract of *Eupatorium adenophorum.* Abbreviations: CK–control; L–treated by aqueous extract in low concentration; M–treated by aqueous extract in medium concentration; H–treated by aqueous extract in high concentration.

Three metabolites involved in energy metabolism, (ribulose-5-phosphate, alpha-ketoglutaric acid, and glucose-1-phosphate) presented a declining trend when treated with *E. adenophorum* aqueous extract (Figure 7). Comparing L and CK, there was no significant change in ribulose-5-phosphate content. In the M group, ribulose 5-phosphate showed a decrease. When treated with high-concentration extract, ribulose-5-phosphate was almost undetectable (Figure 7). As to alpha-ketoglutaric acid, there was a clear declining trend with the increase of concentration of *E. adenophorum* extract. For glucose-1-phosphate, *E. adenophorum* extract induced downregulation, but this was not concentration dependent. In general, *E. adenophorum* extract negatively influenced three essential substances in energy metabolism.

**Figure 8:**
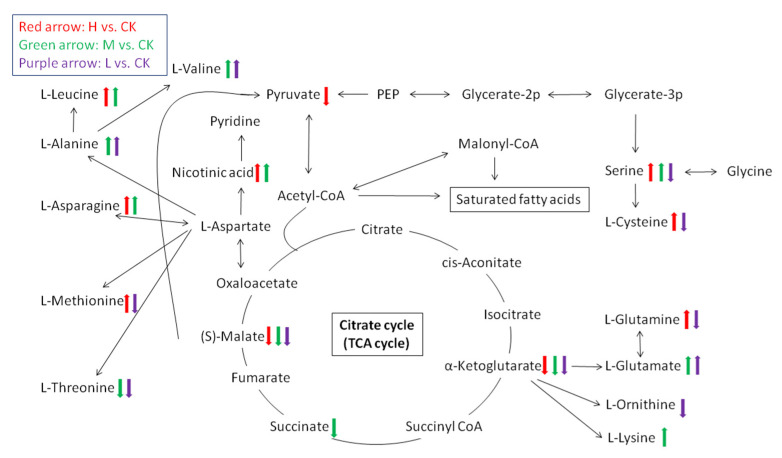
Schematic overview of the main impacted metabolites and major metabolic pathways related to energy and amino acid metabolism in *Arabidopsis thaliana*. Upward arrows represent upregulation, and downward arrows represent downregulation. Three colors of arrows represent three treatments of *Eupatorium adenophorum* aqueous extracts.

Figure 8 shows a schematic overview of the main impacted metabolites and their relationship in energy and amino acid metabolism pathways. As mentioned above, for the citrate cycle (TCA cycle), two key substance, (S)-Malate and alpha-ketoglutarate were downregulated after *A. thaliana* exposure to *E. adenophorum* extracts of three different concentrations (H, M, and L). Figure 8. Shows that the citrate cycle had various relationships with amino acid contents. (S)-Malate had a relationship with serine and cysteine. Alpha-ketoglutarate had relationships with glutamine, ornithine, and lysine. Besides, several amino acids, such as valine, leucine, alanine, asparagine, methionine and threonine were linked with L-aspartate, which had a mutual transformation with oxaloacetate in the TCA cycle (Figure 8). Overall, the contents of most amino acids increased when treated with high and medium concentration extracts (H and M group). Only threonine, cysteine, glutamine, and ornithine showed downregulation in the low concentration extract (L group). The accumulation of many amino acids indicated that nitrogen metabolism was affected by allelopathy or that there was significant protein degradation.

### 2.3. Phosphate Absorption Experiment

**Figure 9:**
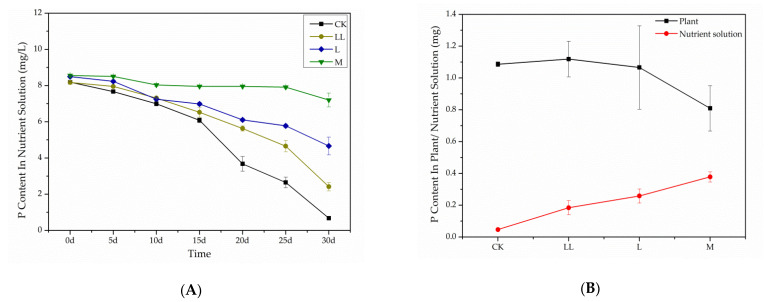
Changes in phosphate concentration in nutrient solution over time (**A**), and changes of total phosphorus in *Arabidopsis thaliana* plants treated with *Eupatorium adenophorum* aqueous extracts and the residual phosphorus in nutrient solution (**B**). Abbreviations: CK–control; LL–treated by 6.25% aqueous extract; L–treated by 12.5% aqueous extract; M–treated by 25% aqueous extract. Error bars represent standard error (SE).

Based on the results of metabolomic analysis, mineral absorption might be significantly impacted after *E. adenophorum* extract exposure, especially the absorption of phosphate (Figure 5 and Figure 6). To verify this conclusion, phosphate absorption of *A. thaliana* was tested. After 30 days of hydroponics with *A. thaliana* plants, the concentration of phosphate in the nutrient solution of CK decreased by 91.7%, while phosphate in the LL solution (Hoagland solution with 6.25% *v/v* aqueous extract) and L solution (Hoagland solution with 12.5% *v/v* aqueous extract) decreased by 70.5% and 45.1%, respectively. In the M solution (Hoagland solution with 25% *v/v* aqueous extract), phosphate content showed very little decrease during the 30 day incubation (Figure 9A). These results indicate that *E. adenophorum* extracts prevented the absorption of phosphorus by *A. thaliana* seedlings. The following test of phosphorus content in *A. thaliana* plant tissues further demonstrated these results. The phosphorus content in *A. thaliana* tissues exposed in M solution was much lower than that in the CK, LL and L solutions. The residual phosphate in the M solution was much higher than that in L, LL and CK solutions (Figure 9B). These results in the hydroponic experiment demonstrated that mineral absorption was an important pathway affected by allelochemicals in *E. adenophorum* extracts.

## 3. Discussion

*Eupatoriumadenophorum* is not only an invasive species but also an allelopathic plant [20]. Laboratory bioassays usually offer clear evidence on phytotoxicity of secondary compounds released by invasive plants. Our in vitro bioassay also showed significant inhibitory effects on the recipient species, *A. thaliana* (Figure 1 and Figure 2). There are several reasons why we did the bioassay with aqueous extracts rather than pure isolated allelochemicals. First, we wanted to simulate the leaching process of allelochemicals in natural settings. Second, the components of *Eupatorium* aqueous extract have been detected and demonstrated by previous studies [9,14,21], and thus the experiment would be repeatable. Finally, according to our experience, Euptox A, a main allelochemical in *Eupatorium* aqueous extract, is not easily dissolvable in water under laboratory conditions. The allelochemicals leached by rains and fog drips enter the soil in natural ecosystems, and part is degraded by soil microorganisms [22]. However, our previous research found that even those allelochemicals maintained in extremely low concentrations still had phytotoxic effects [14]. Therefore, the metabolomics experiments with *A. thaliana* was conducted under the same conditions as the laboratory bioassay.

As described in the Results section, three important metabolites in energy metabolic pathways, ribulose-5-phosphate, alpha-ketoglutaric acid and glucose-1-phosphate, showed downregulations. Ribulose 5-phosphate is one of the end-products of the pentose phosphate pathway, and derives from a ribulose. Alpha-ketoglutaric acid is also named as alpha-ketoglutarate, which acts as a key substance and intermediate in the citrate cycle (also known as the TCA cycle), in which stored energy is released by a series of chemical reactions (Figure 8). Glucose-1-phosphate derives from a glucose and plays a role as a fundamental metabolite. Glucose-1-phosphate is metabolized via the oxidative pentose phosphate cycle and the glycolytic pathway, which exist within the chloroplasts of higher plants. Pentose phosphate produces a large amount of phosphoribose and other important intermediates related to photosynthesis and achieves monosaccharide conversion.

Amino acids are a large group of substances involved in plant primary metabolism and play important roles in plant physiological processes. For instance, they can act as osmotic substances, regulate stomatal opening and act as precursors for the synthesis of defense-related metabolites and signaling metabolites [23]. In our study, the results of metabolomic analysis of *A. thaliana* showed a high impact on amino acidic pathways. Several amino acids, such as asparagine, which is involved in osmotic adjustment, had significant increments. Previous research demonstrated that asparagine plays a critical role in the recovery of plants from osmotic stress [24,25]. Because allelopathic compounds, particularly donor plant extracts or leachates, may change osmotic potential, observed changes in this kind of amino acid in our study were easily to understand. Asparagine content significantly increased in *A. thaliana* exposed to *Eupatorium* aqueous extracts, while a significant decrease of its derivative, threonine, was observed. Previous research found that aspartic acid, lysine, and threonine represent building blocks for stress-specific proteins [26,27]. Therefore, the fluctuation of these amino acids in the treated plant, *A. thaliana*, should be a reasonable reaction to biotic stress. Interestingly, distinct increase in leucine, valine and alanine observed in *A. thaliana* generally occurred under protein degradation, as in aged leaves before abscission or in plant cell resting cultures [28]. In particular, the increase of serine content indicated that plants experienced changes in photorespiration rate, and the photorespiratory cycle, as demonstrated by Bourguignon et al. [29]. Glycine (Gly) and serine (Ser) are two essential amino acids formed during photorespiration, and the Gly/Ser ratio is commonly used as an indicator of photorespiratory activity [30].

It is worth mentioning that although carbohydrate metabolism was also affected by allelochemicals, there was no consistent trend of changes in sugars and their derivatives. Some sugars and derivatives, such as ribose, L-threose, tagatose and 2-deoxy-D-galactose, showed declining trends compared to the control. However, sedoheptulose, trehalose, and gluconic lactone had rising trends compared to the control (Table S1). Glucose was increased during treatments with low and medium concentration (L and M) aqueous extracts, but suppressed by the high concentration (H) aqueous extract. Taken together, these results suggest that biosynthetic phases of photosynthesis were affected by *E. adenophorum* extracts, and disorders of sugars metabolism occurred.

It is also notable that pyruvate showed a significant decline in the high concentration treatment (H) (Figure 8). Pyruvate is an intermediate compound in the metabolism of carbohydrates, proteins and fatty acids. We can infer that other metabolic pathways, including sugar metabolism, protein synthesis and decomposition, and lipid metabolism, were also affected by allelopathy.

Previous phytochemical analysis of *E. adenophorum* demonstrated the strong presence of a sesquiterpene, Euptox A, which was subsequently known as the main effective allelochemical of this noxious plant. The sesquiterpenes are a set of compounds that can cause oxidative stress and a cascade effect on the physiological processes of the recipient plants, and can influence the receptor plant’s development and growth [31]. Sesquiterpenes can destroy cell membrane integrity, affect mitochondrial respiration and microtubule distribution [32,33]. Sesquiterpenes can also change plant hormone and water status and inhibit seed germination and seedling growth of several plants including *A. thaliana* [34,35]. Hussain et al. [36] revealed that the terpenoid artemisinin could not only affect photosynthetic efficiency but also result in oxidative stress and lipid peroxidation in plant roots, as well as impact on calcium and nitrogen absorption in *A. thaliana*. In our study, phosphate absorption was the pathway primarily affected by *Eupatorium* aqueous extract, indicating a possibility of destruction of the cell membrane and phosphorus carrier protein. Phosphate transportation is closely associated with root hairs. So, further research should pay more attention to the microstructure of the root tips of the receptor plants.

## 4. Conclusions

In this study, the allelopathic potential of *E. adenophorum* was confirmed through an in vitro bioassay, using aqueous extracts that imitated natural leached allelochemicals. Negative effects on the growth of the recipient plant, *A. thaliana*, were observed and these inhibitory effects were concentration-dependent, consistent with previous studies [21]. Most importantly, biochemical evidence of phytotoxicity of this invasive plant, *E. adenophorum*, was found by the method of Gas Chromatographic−Mass Spectrometric detection and metabolomic analysis. Energy metabolism, especially of the citrate cycle (TCA cycle), was negatively affected by allelopathy. Amino acidic pathways were highly impacted by *E. adenophorum* extract, which resulted in an increment of most amino acids when *A. thaliana* was treated by the high concentration extract. In addition, mineral absorption of the recipient plant was significantly suppressed. The hydroponic experiment in our study demonstrated that *E. adenophorum* allelochemicals reduced phosphate absorption of *A. thaliana*. In conclusion, *E. adenophorum* is an allelopathic species that can inhibit nearby plant species by disordering their metabolism, thus helping its invasion.

## 5. Materials and Methods

### 5.1. Plant Material Collection and Aqueous Extracts Preparation

In November of 2017, fresh aerial parts of *E. adenophorum* were collected from a natural population in Panzhihua (Sichuan Province in Southwest China). The leaves and stems were rinsed with tap water to remove dust and their surfaces were sterilized with 0.3% (*v/v*) NaClO for 15 min, followed by three washes by distilled water. Leaves were immediately soaked in distilled water (1 g fresh material: 10 g distilled water) for 36 h at room temperature [37], then the leaves and stems were removed, and the aqueous extract was filtered using a 0.45 μm syringe filter to remove microorganisms. *A. thaliana* seeds (col-0) were purchased from South China Agricultural University (Guangdong Province in Southeast China). *A. thaliana* seeds were surface sterilized with 0.3% hydrogen peroxide for 5 min and rinsed three times with distilled water.

### 5.2. In Vitro Bioassay

The bioassay experiment was conducted in November 2017. MS medium (Murashige & Skoog medium) powder containing sucrose and agar was purchased from Solarbio Co. Ltd., Its nutrient content can be found at www.solarbio.net (2 November 2017). MS medium powder was dissolved in an appropriate amount of water and autoclaved for 45 min at 121 °C. Sterilized MS medium, aqueous extract, and distilled water were mixed in an appropriate proportion to make a “testing medium”. The prepared “testing medium” with 50% aqueous extract was labeled as H (medium with high concentration extract); the medium with 25% aqueous extract was labeled as M (medium with medium concentration extract), and the medium with 12.5% aqueous extract was labeled as L (medium with low concentration extract). Another MS medium was prepared using pure distilled water, which was labeled as CK. All the media had natural pH. *A. thaliana* seeds were vernalized in a 4 °C refrigerator for 48 h before in vitro bioassays. Thirty seeds were sowed in 9 cm-diameter Petri dishes containing the solid media described above. Six replications for each testing medium were conducted. All 24 Petri dishes were placed randomly in a 50% humidity incubator (LEAD-Tech EHI 300) with 16 h light (10,000 Lux) at 28 °C and 8 h dark at 20 °C as a cycle. All the seeds were incubated for 27 days, then rosette leaf length of the emerged plants was measured by a vernier caliper. The rosette leaf length was defined as half of the canopy diameter of the plant. After that, the fresh plants were excavated from the media, washed with distilled water and drained with filter paper. The root length was measured, the fresh biomass was weighed, then the plants were dried at 65 °C for 48 h, and dry weight recorded. All data were normally distributed. One-way ANOVAs were conducted on root length, rosette leaf length, fresh and dry weight of all plants in each Petri dish with aqueous extract treatment as the fixed factor. Multiple comparisons were conducted according to Tukey’s Honest Significant Difference (HSD) test.

### 5.3. Metabolomic Experiment

An experiment the same as the bioassay described above was conducted at the same laboratory in November 2017. All *A. thaliana* seeds were cultivated for 27 d, which was consistent with the bioassay. Thereafter, *A. thaliana* seedlings were harvested immediately for metabolite analysis (seedlings in a petri dish were considered to be one sample). The plant samples were processed with a method modified from Zhang et al. [38]. Gas chromatography−mass spectrometry (GC-MS) detection and data analysis were conducted as in Zhang et al. [38]. 

#### 5.3.1. GC-MS Sample Preparation

For each sample, fresh plant material was ground into powder in liquid nitrogen and 60 mg plant powder was transferred to a 1.5 mL Eppendorf tube. Two small steel balls were added to the tube. 360 μL of cold methanol and 40 μL of 2-chloro-l-phenylalanine (0.3 mg/mL) dissolved in methanol as internal standards were added to each sample. All the samples were placed at -80 °C for 2 min and then sonicated at 60 HZ for 2 min. Chloroform (200 μL) was added to each sample, vortexed, then 400 μL of water was added and the sample vortexed again. Thereafter the samples were ultrasonicated at ambient temperature for 30 min. The samples were centrifuged at 12,000 rpm for 10 min at 4 °C. A quality control (QC) sample was prepared by mixing aliquots of all samples. An aliquot of the 300 μL supernatant was transferred to a glass sampling vial for vacuum drying at room temperature, then 80 μL of 15 mg/mL methoxylamine hydrochloride in pyridine was added. The resultant mixture was vortexed vigorously for 2 min and incubated at 37 °C for 90 min. N, O-bis(trimethylsilyl)trifluoroacetamide (80 μL, BSTFA with 1% trimethylchlorosilane), and 20μL n-hexane were added into the mixture, then vortexed vigorously for 2 min and derivatized at 70 °C for 60 min. The samples were placed at ambient temperature for 30 min before GC-MS analysis.

#### 5.3.2. Gas Chromatographic−Mass Spectrometric Analysis

The derivatized samples were analyzed on an Agilent 7890B gas chromatography system coupled to an Agilent 5977A MSD system (Agilent Technologies Inc., Santa Clara, CA, USA). A DB-5MS fused-silica capillary column (30 m × 0.25 mm × 0.25 μm, Agilent J & W Scientific, Folsom, CA, USA) was utilized to separate the derivatives. Helium (>99.999%) was used as the carrier gas at a constant flow rate of 1 mL/min through the column. The injector temperature was maintained at 260 °C. The injection volume was 1 μL in splitless mode. The initial oven temperature was 60 °C, ramped to 125 °C at a rate of 8 °C/min, to 210 °C at a rate of 4 °C /min, to 270 °C at a rate of 5 °C/min, to 305 °C at a rate of 10 °C/min, and finally held at 305 °C for 3 min. The temperature of the MS quadrupole and ion source (electron impact) was set to 150 and 230°C, respectively. The collision energy was 70 eV. Mass data were acquired in a full-scan mode (m/z 50–500), and the solvent delay time was set to 5 min. The QC samples were injected at regular intervals (every 12 samples) throughout the analytical run to provide a set of data from which could be assessed repeatability.

#### 5.3.3. Data Processing

ChemStation (version E.02.02.1431, Agilent, Santa Clara, CA, USA) software was used to convert the raw data (D format) to CDF format, and then CDF data were imported into the ChromaTOF software (version 4.34, LECO, St Joseph, MI) for data processing. Metabolites were identified by the Fiehn database, which was linked to ChromaTOF software. The data were normalized to the total peak area of each sample, multiplied by 10,000 and transformed by log2. According to the matching of mass spectrometry fingerprint and gas chromatography retention time, the total amount of metabolites was identified and semiquantified. GC-MS detected 715 peaks and the original mass spectrometry data detected 234 substances. Unsupervised principal component analyses (PCA) and the supervised partial least-squares discriminant analysis PLS-DA clustering method were performed on GC-MS data via online resources (http://www.metaboanalyst.ca/) (10 January 2019). Metabo Analyst was used to perform unsupervised clustering principal component analysis (PCA) on GC-MS data, which provided clustering information between groups. The grouping of samples in the PCA scale was based on the similarity of metabolic characteristics of the samples or treatments. To isolate the differences among the groups to the greatest extent, we used PLS-DA multivariate analysis based on GC-MS data sets. Variable importance in projection (VIP) was determined as the weighted sum of squares of the (O)PLS-DA analysis, which indicated the overall contribution of each variable to the OPLS-DA model. Those variables with VIP > 1 were considered relevant for separation and defined as discriminating metabolites. The differential metabolites were selected based on the combination of a statistically significant threshold of VIP values larger than 1.0 and *p* values less than 0.05 from a two-tailed Student’s *t*-test. Biological pathway analysis was performed on GC-MS data using Metabo Analyst 2.0. The impact value threshold calculated for pathway identification was set at 0.1.

### 5.4. Phosphate Absorption Experiment

In February 2019, a hydroponic experiment was conducted to examine phosphate absorption of *A. thaliana* exposed to *E. adenophorum* aqueous extracts. Plant material of *E. adenophorum* were collected from the same place in Panzhihua in November 2018. Fresh aqueous extract was made and sterilized by the method described above. The nutrient powders for Hoagland nutrient solutions were dissolved in a proper amount of water and autoclaved (SANYO MLS-3750 autoclave) for 30 min at 121 °C. Then, we mixed the nutrient solution, the original aqueous extract, and distilled water in a proper proportion to make “testing Hoagland solutions”. The testing solution with 25% (*v/v*) aqueous extract was labeled M (medium concentration). The testing solution with 12.5% (*v/v*) aqueous extract was labeled L (low concentration). The testing solution with 6.25% (*v/v*) aqueous extract was labeled LL (lower concentration). Hoagland nutrient solution prepared by pure water was labeled as CK. All the solutions were at natural pH. *A. thaliana* seeds were grown in MS medium for 20 days before the hydroponic experiment. *A. thaliana* seedlings of uniform growth were then transplanted to a 50 mL bottle containing M, L, LL and CK Hoagland testing solutions. Each treatment had four replications. Phosphorus contents in the nutrient solutions were measured with the Mehlich 3 (M3) method every five days for the successive 30 days [39]. Thereafter *A. thaliana* plants were harvested, all the organs from each plant were mixed, and phosphorus contents in the plant tissues were determined using ICP-AES [40].

## Data Availability

Data is contained within the article or Supplementary Material.

## Supplementary Materials

The following are available online at https://www.mdpi.com/article/10.3390/plants10071473/s1, Table S1: Differential metabolites impacted by L, M and H extract solutions. Red represents up regulation, green represents down regulation, yellow represents no consistent regulation in different groups.
