# Supplementary material for: Metabolomics Reveals the Allelopathic Potential of the Invasive Plant Eupatorium adenophorum
Source: Plants (Basel). 2021 Jul 19;10(7):1473. doi: 10.3390/plants10071473 (PMC8309375; doi:10.3390/plants10071473)
Supplement: Supplementary file 1 [file plants-10-01473-s001.zip › plants-1265750-supplementary.pdf]

Table S1. Differential metabolites impacted by L, M and H extract solutions. Red represents up regulation, green represents down regulation, yellow represents no consistent regulation in different groups.

a(27)	b(12)	c(12)	d(45)	e(15)	f(9)	g(25)
tyrosine	valine	D-alanyl-D-alanine	(S)-Mandelic acid	3-hydroxybutyric acid	citrulline	glutamic acid
1-Hydroxyanthraquinone	threonine	ornithine	1-Methylhydantoin	3-Hydroxynorvaline	glutamine	N-Acetyltryptophan
2-Indanone	Isoleucine	2-Deoxytetronic acid	22-Ketocholesterol	8-Aminocaprylic acid	L-cysteine	proline
2-ketobutyric acid	L-glutamic acid	sorbitol	2-Deoxyerythritol	Acetol	methionine	maleic acid
Analyte 677	alanine	Glucose-1-phosphate	2-Furoic Acid	adenosine	2-Deoxy-D-galactose	malonic acid
ascorbate	fructose	Gallic acid	3-Cyanoalanine	alpha-ketoglutaric acid	D-Talose	urea
benzamide	Levoglucosan	2-aminoethanethiol	3-hydroxy-3-methylglutaric acid	Cellobiotol	maltose	Pyruvic acid
Benzoylformic acid	glycerol	adenine	3-hydroxy-L-proline	creatine degr	Tagatose	Gentiobiose
beta-Hydroxymyristic acid	Ethanolamine	2-Monoolein	5,6-Dimethylbenzimidazole	D-galacturonic acid	O-Phosphorylethanolamine	Glucoheptonic acid
carbamoyl-aspartic acid	cycloleucine	Leucrose	5-Aminovaleric acid	Dithioerythritol		glucose
Carbobenzyloxy-L-leucine degr2	xylose	Malonamide	5-Hydroxyindole-2-carboxylic acid	L-Malic acid		Sedoheptulose
Cumic Acid	Isopropyl-beta-D-thiogalactopyranoside	Purine riboside	5-Methoxytryptamine	maltotriose		N-Methylanthranilic acid
Digalacturonic acid			Acetophenone	phosphate		2-amino-2-methylpropane-1,3-diol
Ethyl cinnamate			Alizarin	quinic acid		pyrogallol
indole-3-acetamide			Allantoic acid	serine		indole-3-acetic acid
inosine			alpha-D-glucosamine 1-phosphate			Dodecanol
L-homoserine			Aminooxyacetic acid			ribulose-5-phosphate

lysine
N-Acetyl-beta-D-mannosamine
naringenin
Norleucine
oxamide
panthenol
saccharopine
succinic acid
uracil
xylitol

asparagine
beta-Mannosylglycerate
Carbobenzyloxy-L-leucine
D-erythronolactone
D-erythro-sphingosine
dibenzofuran
L-Allothreonine
L-dopa
leucine
Maleamate
Maleimide
methyl octanoate
Mono(2-ethylhexyl)phthalate
N-Carbamylglutamate
N-Ethylglycine
nicotinic acid
Nicotinoylglycine
noradrenaline
norvaline
oxalic acid
oxoproline
palatinitol
p-benzoquinone
Phenyl beta-D-glucopyranoside
Phytanic acid

(2R,3S)-2-hydroxy-3-isopropylbutanedioic acid
2,3-Dihydroxypyridine
2,6-Diaminopimelic acid
2-hydroxypyridine
Analyte 731
D-Glyceric acid
epsilon-Caprolactam
N-Acetylisatin

	phytosphingosine
	thymine
	trehalose
